# Role of the COP9 Signalosome (CSN) in Cardiovascular Diseases

**DOI:** 10.3390/biom9060217

**Published:** 2019-06-05

**Authors:** Jelena Milic, Yuan Tian, Jürgen Bernhagen

**Affiliations:** 1 Vascular Biology, Institute for Stroke and Dementia Research (ISD), Klinikum der Universität München (KUM), Ludwig-Maximilians-University (LMU), 81377 Munich, Germany; jelena.milic@med.uni-muenchen.de (J.M.); yuan.tian@med.uni-muenchen.de (Y.T.); 2 Munich Heart Alliance, 80802 Munich, Germany; 3 Munich Cluster for Systems Neurology (SyNergy), 81377 Munich, Germany

**Keywords:** atherosclerosis, constitutive photomorphogenesis 9 (COP9) signalosome (CSN), cardiovascular diseases, deNEDDylation, heart failure, inflammation, JAB1, myocardial infarction, stroke

## Abstract

The constitutive photomorphogenesis 9 (COP9) signalosome (CSN) is an evolutionarily conserved multi-protein complex, consisting of eight subunits termed CSN1-CSN8. The main biochemical function of the CSN is the control of protein degradation via the ubiquitin-proteasome-system through regulation of cullin-RING E3-ligase (CRL) activity by deNEDDylation of cullins, but the CSN also serves as a docking platform for signaling proteins. The catalytic deNEDDylase (isopeptidase) activity of the complex is executed by CSN5, but only efficiently occurs in the three-dimensional architectural context of the complex. Due to its positioning in a central cellular pathway connected to cell responses such as cell-cycle, proliferation, and signaling, the CSN has been implicated in several human diseases, with most evidence available for a role in cancer. However, emerging evidence also suggests that the CSN is involved in inflammation and cardiovascular diseases. This is both due to its role in controlling CRLs, regulating components of key inflammatory pathways such as nuclear factor kappa-light-chain-enhancer of activated B cells (NF-κB), and complex-independent interactions of subunits such as CSN5 with inflammatory proteins. In this case, we summarize and discuss studies suggesting that the CSN may have a key role in cardiovascular diseases such as atherosclerosis and heart failure. We discuss the implicated molecular mechanisms ranging from inflammatory NF-κB signaling to proteotoxicity and necrosis, covering disease-relevant cell types such as myeloid and endothelial cells or cardiomyocytes. While the CSN is considered to be disease-exacerbating in most cancer entities, the cardiovascular studies suggest potent protective activities in the vasculature and heart. The underlying mechanisms and potential therapeutic avenues will be critically discussed.

## 1. The COP9 Signalosome: Discovery, Structure, and Functions

The constitutive photomorphogenesis 9 (COP9) signalosome (CSN)is an evolutionarily conserved multi-protein complex with a molecular size of ~400 kDa that is found in all species and kingdoms of life, including plants, fungi, *Caenorhabditis elegans*, *Drosophila melanogaster*, and mammals [1,2,3,4,5]. Several excellent articles in this Special Issue cover aspects of CSN biology and pathobiology in plants and fungi. In mammals, the complex consists of eight subunits termed CSN1–CSN8, numbered according to their molecular size [6]. The CSN has multiple functions in the cell. It is involved in cell cycle control, DNA repair, and inflammatory gene expression, among other effects, by functioning both as an enzyme controlling ubiquitin-dependent protein degradation processes and as a docking platform for several kinases and other cell-regulatory proteins [1,2,3,4,5]. In addition, some of the individual CSN subunits have been ascribed complex-independent activities by distinct protein-protein interactions [7]. 

The COP9 signalosome complex was first purified in 1996 by Chamovitz et al. from *Brassica oleracea* (cauliflower). Since it was found to act as a repressor of dark-grown growth patterns, which is similar to other protein complexes in plants such as COP1 that were discovered earlier, it was termed constitutive photomorphogenesis 9(COP9) [8]. Mammalian CSN complexes were isolated two years later from human erythrocytes and pig spleen [9,10]. Subunit 5 of the CSN (CSN5) was identified independently as c-Jun-activation-domain-binding protein-1 (JAB1) and acts as a co-activator of activator protein-1 (AP-1)-regulated gene transcription to stabilize complexes of the transcription factors c-Jun or JunD at their specific AP-1 binding sites, which increases the specificity of target gene activation [11]. Thus, CSN5/JAB1 can exist independently of the holo-complex, but for CSN subunits 2, 4, and 7, functions outside of the holo-complex or as part of smaller sub-complexes have also been suggested [12,13,14,15,16]. The CSN complex has sequence and structural homologies to the 19S lid sub-complex of the 26S proteasome [3,17,18,19]. Subunits CSN1, CSN2, CSN3, CSN4, CSN7, and CSN8 harbor a PCI (proteasome, COP9 signalosome, translation initiation factor) domain, while CSN5 and CSN6 contain an MPN (Mpr1p-Pad1-N-terminal) domain [20,21]. 

The enzymatic activity of the CSN is harbored in CSN5 and is mediated by the Zn^2+^-containing JAMM (JAB1-MPN-domain metalloenzyme) motif of this subunit [22]. It serves to catalyze the cleavage of ubiquitin-like neural precursor cell-expressed developmentally downregulated 8 (NEDD8) conjugates from the cullin subunit of cullin-RING E3 ligases (CRLs), including S-phase kinase-associated protein 1 (SKP1)-CUL1/RBX1-F-box (SCF)-type CRLs, containing cullin-1 (CUL-1) and RING protein ring-box 1 (RBX1) as the enzymatic core, SKP1 as an adaptor, and one of numerous substrate-binding F-box proteins [23]. NEDD8 is an ubiquitin-like protein modifier [24,25,26] and CSN5 is the only CSN subunit with catalytic deNEDDylase activity, which it can only exert in the three-dimensional structural context of the CSN holo-complex [26]. The ubiquitin ligase activity of CRLs depends on its post-translational modification by NEDD8 conjugation, which is bound via an iso-peptide bond. The CSN complex, thus, inhibits the E3 ligase activity of CRLs by removing NEDD8 from the cullin adaptor core [3,27,28,29]. Accordingly, the CSN has an impaired binding affinity to deNEDDylated CRLs and is rapidly ejected from the CSN-CRL complex, whereas binding of the CSN to NEDDylated CRLs is favored [30,31]. Of note, the assembly and disassembly of CRLs has been suggested to be controlled by the NEDDylation and deNEDDylation state of their cullin component, and, thus, by the CSN, as well as by binding of CAND1 [3,23,26,31,32,33,34]. In fact, deNEDDylation by the CSN is a prerequisite for CAND1 binding and for re-modelling of CRLs.

The crystal structure of the human CSN complex was resolved at a resolution of 3.8 Å in 2014 [17]. The structure elucidated the molecular architecture of the complex in stunning detail and provided valuable information regarding the molecular mechanism underlying the interaction between the CSN and CRLs and the regulation of the enzymatic activity of the CSN. While it confirmed some of the conclusions drawn from earlier biochemical, electron microscopy, and modelling studies [13,18], it also held surprises. The structure showed that the eight-subunit CSN complex has two organizational centers. A horseshoe-shaped ring is formed by its six PCI domain proteins and a large bundle is formed by the carboxy-terminal α-helices of each subunit. The MPN domain-containing subunits CSN5 and CSN6 are situated at the core of the bundle. In his News & Views article accompanying the crystal structure publication, Deshaies compares the structure of the CSN with ‘a splayed hand and a tomato’ *(“….a widely splayed hand on which a small box sits askew, topped by a tomato…..Like a hand, the CSN has five digits (the ... ends of CSN1, 2, 4, 7, and 3 plus 8) projecting from an organizing center, the palm. The palm is formed by the ‘winged-helix’ subdomains…., which associate to form a horseshoe-shaped structure. Resting on the hand is the box, formed by…the carboxy-terminal ends of each subunit. Sitting atop…is the CSN5–CSN6 tomato….”* [35]). The structure also revealed that in the absence of substrate (i.e., CRL) binding, the catalytically active site in CSN5 is auto-inhibited by glutamate residue 104 that blocks the Zn^2+^ atom in the active site, which prevents binding of the hydrolytic water molecule. The inactive state is only released upon binding of the NEDDylated CRL substrate, which elicits conformational changes mediated via subunits CSN4 and 6 that eventually lead to the activation of the isopeptidase activity in CSN5 and, therefore, NEDD8 removal from cullins. Thus, the isopeptidase activity of CSN5 within the CSN complex is regulated differently than in other JAMM-containing enzymes such as AMSH-LP and Rpn11 [17,35,36,37].

Cullin-RING ubiquitin ligases are the largest family of E3 ligases in eukaryotes. CRLs share a common rigid scaffold and a RING domain catalytic subunit, but use different adaptors and a plethora of substrate receptors to accommodate unique E3 molecular ubiquitination machines, which allow them to catalyze protein ubiquitination under distinct cellular contexts in response to various signals. By recruiting specific substrate receptor F-box proteins, CSN-controlled CRL-type E3 ligases such as the SCF-type CRLs (CRL1) catalyze the ubiquitylation and proteasomal degradation (jointly referred to as the ubiquitin-proteasome system, UPS) of numerous proteins with key functions in cell cycle regulation, cell proliferation, DNA damage, and inflammatory signaling pathways [26,38,39,40,41,42]. In the context of this review article, substrate receptor F-box protein/‘to-be-degraded-substrate’ pairs related to cell cycle progression, inflammatory signaling, or hypoxic stress such as SCF^β-TRCP1^/ΙκB-α, SCF^β-TRCP1^/Wee1, SCF^β-TRCP1^/Cdc25A, Cul3^KEAP1^/Nrf2, SCF^Skp2^/p27, or Cul2^VHL^/HIF-1α, respectively, are of particular interest [39,43]. As outlined in the subsequent chapters, the degradation of these substrates may directly link the CSN/CRL machinery to inflammatory and stress pathways such as nuclear factor (NF)-κB signaling, hypoxia-inducible factor (HIF)-1α signaling, and cell survival responses, controlling e.g., atherosclerotic plaque inflammation, plaque macrophage, or smooth muscle cell (SMC) proliferation, or cardiac fibroblast hyperactivation, and, thus, playing a role in cardiovascular pathologies. 

Moreover, the CSN regulates other E3 ubiquitin ligase classes such as MDM2 (mouse double minute 2 homolog) and COP1 (constitutively photomorphogenic 1) that target similarly relevant inflammatory proteins such as p53, 14-3-3σ, or c-Jun [44,45,46]. The CSN was also shown to bind USP15, which is a deubiquitinase (DUB) involved in NF-κB signaling [47]. By serving as a docking platform for kinases such as casein kinase II (CK-2) or protein kinase D (PKD), the CSN also co-controls the phosphorylation of NF-κB and c-Jun, as well as that of certain CSN subunits themselves [9,48]. By directly interacting with key inflammatory and apoptosis mediators such as macrophage migration inhibitory factor (MIF), p53, c-Jun, Smad4, or the β2 integrin LFA1, possibly in a CSN complex-independent manner, subunit CSN5/JAB1 controls inflammatory, fibrogenic, and tumorigenic pathways [11,49,50,51]. The interaction between CSN5 and c-Jun or JunD also exemplifies direct functions of the CSN in controlling gene expression. As mentioned above, CSN5 stabilizes AP-1 and enhances AP-1 activity at AP-1 promoter sites and CSN5 (and CSN2) promote the JNK/AP-1 pathway in several ways [11,49,50], while CSN1 has been reported to suppress AP-1 activity [52]. These functions are also relevant in atherosclerosis (see Section 2).

Due to its critical control function of the UPS pathway, its role as a docking platform for cell-regulatory enzymes and, due to the various individual functions of some of its subunits, it is clear that the CSN is an integral molecular machinery guaranteeing cell homeostasis, cell survival, and normal physiological cell function at several levels. Thus, this generally plays a protective role in healthycells. However, when dysregulated, the multiple mechanisms through which the CSN controls key cellular pathways also predispose this complex as a major target in human disease. To date, most evidence has been gathered on the role of the CSN in cancer. In part due to the pivotal cellular role of the above-mentioned CRL substrates in the cell cycle and in apoptosis, but also based on the notion that CSN5 as well as a couple of the other subunits were found to be markedly upregulated in various tumor tissues [53,54,55]. Of note, the pharmacological inhibitor MLN4924 (Pevonedistat, 1S,2S,4R)-4-(4-(((S)-2,3-dihydro-1H-inden-1-yl)amino)-7H-pyrrolo [2,3-d]pyrimidin-7-yl)-2-hydroxycyclopentyl)methyl sulfamate) has been in clinical trials for patients with advanced nonhematological malignancies and acute myeloid leukemia and myelodysplastic syndromes [56,57,58]. MLN4924 inhibits cullin NEDDylation by inhibiting the NEDD8-activating enzyme E1 (NAE) [56]. MLN4924 activity, therefore, somewhat mirrors the deNEDDylation activity of CSN5/CSN, even though off-target effects of MLN4924 have been discussed as well.

A role of the CSN in other diseases including inflammation and heart diseases is emerging [59] and, moreover, recent efforts have identified additional compound categories with higher and/or more distinct specificities such as CSN5i-3 [43], which directly binds to CSN5 and inhibits its deNEDDylation activity, which results in an increased portion of active NEDDylated CRLs, or DCN1 that targets multi-protein NEDD8-E3 ligase complexes by interfering with the N-terminal acetylation–dependent interaction of UBE2M (the E2-conjugating enzyme for NEDD8) with DCN1 (the E3 ligase for NEDD8) and, thereby, blocking NEDD8 ligation to cullin proteins [60]. Both of these compounds have already shown utility in preclinical cancer models by underscoring that the CSN and/or its deNEDDylation activity and CRL-controlling functions are a promising druggable target.

**Figure 1:**
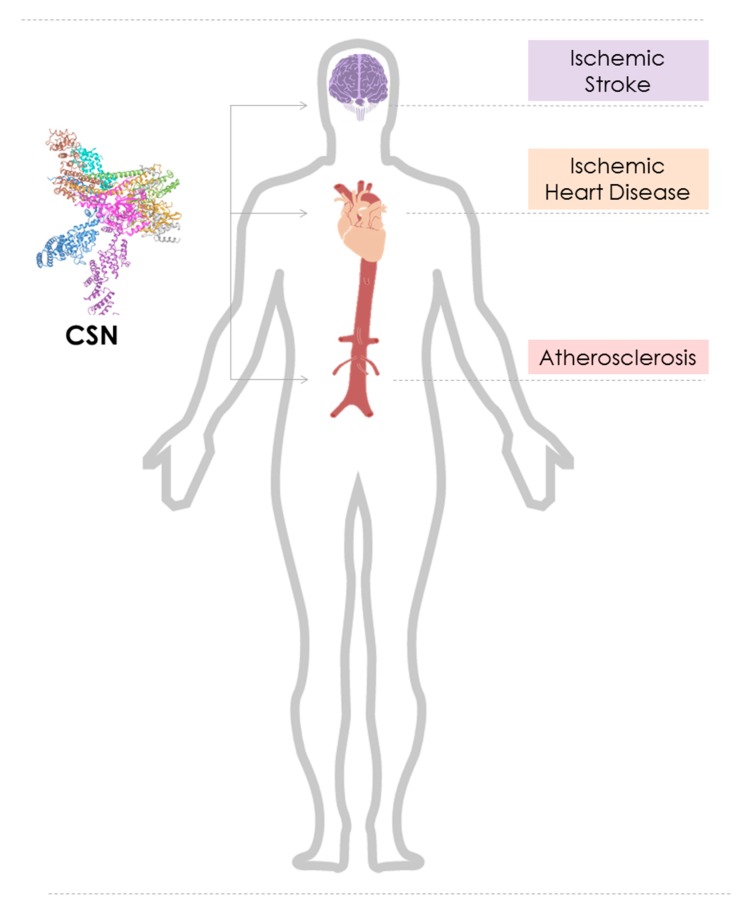
Graphical representation of the role of the constitutive photomorphogenesis 9 (COP9) signalosome (CSN) in cardiovascular diseases. Cartoon illustrating the cardiovascular diseases atherosclerosis, ischemic heart disease, and ischemic stroke, the affected organs/tissues (aorta/vasculature (red), heart (beige/orange), brain (magenta), respectively, and their locations in the human body. The emerging role of the CSN (PDB ID: 4D10) in these diseases is indicated.

In this scenario, we focus on the emerging role of dysregulated CSN function in cardiovascular diseases, namely atherosclerosis, ischemic heart disease (IHD), and heart failure (HF), as well as ischemic stroke (Figure 1). We will briefly introduce the reader into the backgrounds of these diseases, discuss the mechanisms through which the CSN may control pathways contributing to disease pathogenesis, scrutinize the involved cell types and tissues, and discuss potential translational opportunities.

## 2. The Role of the COP9 Signalosome in Atherosclerosis 

Atherosclerosis is a multi-focal, progressive, chronic inflammatory disease of medium and large size arteries that is initially triggered by endothelial dysfunction and activation at locations of low shear stress or disturbed laminar flow (pro-atherogenic predilection sites). Endothelial activation involves the secretion and endothelial deposition of chemokines and upregulation of adhesion molecules, which are key mediators facilitating atherogenic leukocyte arrest and transmigration [61,62,63]. The atherosclerotic lesion is characterized by an accumulation of cholesterol and oxidized lipoproteins, an infiltration of monocytes/macrophages, and an accumulation of foam cells, as well as the proliferation of smooth muscle cells [63,64,65,66,67,68,69]. While the atherogenic process is initially clinically asymptomatic, it evolves over years and decades from a reversible fatty streak stage to advanced fibrous lesions featuring lipid crystals and a necrotic core, which, overall, involves an extensive remodeling of the arterial vascular wall architecture (atherosclerotic plaque formation) [70,71,72]. This is accompanied by luminal stenosis and can, ultimately, lead to plaque instability, plaque rupture, and thrombus formation (luminal thrombosis, atherothrombosis) [73]. If the thrombotic event is occlusive, it can lead to myocardial infarction in the heart and ischemic stroke in the brain [74,75]. Accordingly, atherosclerosis is the main underlying condition of acute myocardial infarction (AMI, heart attack, ischemic heart disease, IHD), ischemic stroke (large artery stroke), and peripheral artery disease (PAD) [64,76,77]. Collectively, cardiovascular diseases (CVD) continue to be the leading cause of morbidity and mortality worldwide, accounting for more than 17 million deaths per year [78]. Furthermore, the ‘inflammatory hypothesis’ of atherosclerosis [79] was recently validated by a large multicenter clinical trial of over 10,000 patients with a history of a previous myocardial infarction and elevated high-sensitivity C-reactive protein (CRP) (the CANTOS trial), which demonstrated that anti-inflammatory therapy targeting the pivotal inflammatory cytokine interleukin-1β (IL-1β) with a monoclonal antibody (called canakinumab) led to a significantly lower rate of recurrent cardiovascular events than the placebo (www.ClinicalTrials.gov, NCT01327846) [80]. However, side effects such as increased lung infections were also noted, which suggest that more research into the inflammatory targets of atherosclerosis is mandated in efforts to better tailor anti-inflammatory therapies in atherosclerotic disease. Among the many cell types that drive the atherosclerotic inflammatory cascade, endothelial cells (ECs), monocytes/macrophages, foam cells, vascular smooth muscle cells (VSMCs), and T cells have been extensively characterized, expressing crucial inflammatory pathways that could be subject to therapeutic targeting. The CSN is involved in several of these pathways and its key role in endothelial, monocyte/macrophage, T-cell, and VSMC inflammation has emerged in the past five to 10 years. Additionally, signaling and transcriptional pathways known to be controlled by the CSN such as NF-κB and AP-1 (see Section 1) are prototypical pathways implicated in atherosclerotic pathogenesis [81,82,83,84,85,86,87,88]. In the following sub-chapters, we will summarize the mechanisms as to how the CSN controls inflammatory signaling pathways in these atherogenic cell types and discuss the nature and potential value of inhibitory strategies that have been pursued in this context.

### 2.1. The COP9 Signalosome Inhibits Atherogenic Signaling Pathways in Endothelial Cells

The activated vascular endothelium responds to inflammatory and atherogenic stress by synthesizing inflammatory products such as chemokines, cytokines, and adhesion molecules [77]. Alterations of the endothelial phenotype into a dysfunctional activated state impose a pathogenic risk factor for several vascular diseases including atherosclerosis [89]. Our previous studies verified that CSN subunit CSN5, as well as subunits CSN1 and CSN8, are highly expressed in the endothelial layer of early human atherosclerotic plaques and their expression was significantly upregulated during the progression of atherosclerosis [90,91,92]. 

The transcription factor NF-κB regulates multiple aspects of innate and adaptive immunity and serves as a pivotal mediator of inflammation, proliferation, and cellular decision-making between apoptosis and survival [54,93,94]. In vascular inflammation and atherogenesis, NF-κB controls the expression of inflammatory cytokines, chemokines, and adhesion molecules that together orchestrate the adhesion and recruitment of leukocytes. For example, inflammatory stimulation of arterial endothelial cells (ECs) with lipopolysaccharide (LPS)/endotoxin, TNF, or IL-1β triggers the NF-κB-mediated upregulation/secretion of inflammatory chemokines such as CCL2, CXCL1, or CXCL8, and vascular adhesion molecules such as vascular cell adhesion molecule (VCAM), intercellular adhesion molecule (ICAM), or E-selectin. In addition, numerous genes that regulate differentiation, survival, and proliferation of vascular and lesional cells involved in the inflammatory response are targets of NF-κB [86]. 

In unstimulated cells, NF-κB exists in the cytoplasm and in a majority of vascular cell types, it forms heterodimers between the p65 and p50 subunit. It is retained in the cytosol by association with the inhibitor of NF-κB, IκB-α, which is a target of the CSN-controlled CRL SCF^β-TRCP1^, as discussed above [54,95,96]. Activation of the NF-κB pathway can be initiated by a large number of extracellular stimuli, which are prototypically inflammatory ones, and predominantly merge into the canonical (classical) NF-κB signaling cascade. For example, inflammatory cytokine receptors such as the tumor necrosis factor receptor (TNF-R), the IL-1 receptor (IL-1R), and toll-like receptors (TLRs, i.e., TLR3, TLR4, or TLR7) are triggered by their corresponding agonists such as TNF, IL-1β, or LPS, respectively. This leads to the downstream activation of the IκB kinase (IKK) complex, which facilitates the ubiquitination, phosphorylation, and proteolysis of IκBα. As discussed in Section 1, K48-dependent polyubiquitination is then carried out by the CRL-type E3 ligase SCF^β-TRCP1^ [96]. Upon degradation of ubiquitinated IκB-α by the UPS, the NF-κB dimer is released to translocate into the nucleus and drive inflammatory gene transcription [54,95,96].

As mentioned in Section 1, the CSN has been generally found to control the NF-κB pathway in a dual manner. Evidence from cell lines and cancer cells showed that, after TNF stimulation, the CSN, via CSN-associated USP15 activity, transiently facilitates the re-accumulation of IκBα, protecting IκBα from sustained UPS-dependent degradation [47]. Moreover, the deNEDDylation activity of the CSN holo-complex itself impairs SCF^β-TRCP1^-mediated ubiquitin conjugation onto IκBα, which results in its stabilization and the attenuation of NF-κB signaling as well [32,47].

We initially studied the relationship between the CSN and NF-κB signaling in human umbilical vein endothelial cells (HUVECs). In line with the studies in cell lines, we observed that silencing of CSN5 by the siRNA approach destabilized the CSN complex and increased the portion of NEDDylated CUL1. This was mirrored by a decrease in IκBα levels and elevated NF-κB activity [90]. Moreover, CSN5 knockdown led to an upregulation of atherogenic chemokines and adhesion molecules and an enhanced the arrest of monocytes on stimulated ECs. The study as well as another report also indicated that high-molecular complexes may form between IKK and the CSN, as shown by co-immunoprecipitation, which dissociate upon inflammatory stimulation [90,97]. Conversely, mimicking CSN5 hyper-activity by applying MLN4924 fully abrogated the inflammatory response in HUVECs, as well as human aortic ECs (HAoECs) and mouse aortic ECs (MAoECs) [91]. The anti-atherogenic effect of MLN4924 in these atherogenic ECs coincided with markedly increased levels of IκB-α and impaired NF-κB transcriptional activity as well as upregulated nuclear levels of the hypoxia-inducible factor (HIF)-1α [91].

Thus, MLN4924, which mimicks CSN5 activity and leads to reduced NEDDylated cullin levels, behaves strongly anti-atherogenic in inflammatory-elicited endothelium. Furthermore, in aggregate, these studies suggest that CSN5 and the CSN exhibit athero-protective activity in the endothelium.

Atherosclerosis starts with endothelial dysfunction, which initiates vascular inflammation but also directly increases endothelial permeability for (oxidized) lipoproteins and other inflammatory mediators. One of the pathways that mediates increased endothelial permeability is the endothelin (ET) receptor/ligand system [98]. The ET system consists of two G protein-coupled receptors: ET type A receptor (ET^A^R) and ET type B receptor (ET^B^R). They can bind to three endogenous ligands: ET-1, ET-2, and ET-3 [99]. Endothelin family agonists are vasoconstrictors and vasopressors. Endothelin-1 is the major endothelial isoform. Its main site of vascular production are endothelial cells [100], which suggests auto-/paracrine activity. It binds to both ET^A^R and ET^B^R [98]. ET^B^ receptors are located predominantly in the lining of the blood vessel wall and are associated with either the release of vasodilators or with receptor clearance of circulating ET-1 [98,101], the latter mainly accounted for by ligand/receptor complex internalization and intracellular degradation. ET-1 has been suggested to contribute to atherosclerosis by acting as a mitogen and by potentiating cytokine release in atherosclerotic lesions, and/or by mediating vascular inflammation and neointima formation. ET-1 overexpression promotes inflammation and lowers high-density lipoproteins (HDL), which results in accelerated progression of atherosclerosis [98,102]. A yeast two-hybrid screen applying an adult human heart cDNA library, as well as pull-down studies in HEK293 cells, provided evidence that Csn5 interacts with ET^B^R as well as with ET^A^R [103]. Overexpression of CSN5 facilitated ubiquitination and degradation of ET^A^R and ET^B^R, and knockdown of CSN5 increased ET^A^R cell surface levels [103]. While the exact mechanism of how CSN5 promotes ETR ubiquitination remains unclear, it has been suggested that the increase in ETR ubiquitination is associated with binding of CSN5 to ETR. Moreover, CSN5 binding favored the ubiquitination and degradation of ET^B^R over ET^A^R, while recruitment of CSN5 to ET^A^R was upregulated by ET-1 agonist binding to its receptor, which promotes ET^A^R ubiquitination and degradation associated with increased ERK1/2 phosphorylation [103]. This study, thus, implies that the stability of the pro-atherogenic endothelial cell receptor ET^A^R and ET^B^R is directly controlled by CSN5, but in vivo evidence shows that this may play a role in atherosclerosis and mechanistic insight shows how ETR engagement of CSN5 leads to receptor degradation. This has remained nebulous. 

### 2.2. The COP9 Signalosome Blocks Inflammatory Signaling in Myeloid Cells to Protect from Atherosclerosis

Myeloid cells are key drivers of the inflammatory response in atherosclerosis [65,66,68,104]. Monocytes arise from proliferating and differentiating hematopoietic stem and progenitor cells in the bone marrow (BM) and extensive BM monocyte production has been reported to contribute to atherogenesis in hypercholesterolemic models [105,106]. More recently, it was shown that hypercholesterolemia also induces monopoiesis in extramedullary organs, including the spleen [105,107]. Mobilization of monocytes from BM and spleen and their trafficking into atherosclerotic lesions prominently depends on a plethora of chemokine/chemokine receptor interactions. Numerous studies have demonstrated the importance of the CCR2/CCL2 (MCP-1), CX3CR1/CX3CL1 (fractalkine), CXCR2/CXCL1, and CCR5/CCL2/CCL5 interactions in the progression of experimental atherosclerosis [108,109,110]. While CCL2 mainly affects monocyte mobilization from the BM, CCL5 and CXCL1 are critical drivers of monocyte recruitment into atherosclerotic lesions [111,112]. A comparison of the extent of atherosclerotic lesion formation between *Cxcr2*-deficient and *Cxcl1*-deficient atheroprone mice, i.e., comparing receptor versus ligand knockouts, prompted studies into potential additional CXCR2 ligands with a role in atherosclerosis. These studies identified the inflammatory cytokine MIF as a novel atypical chemokine that promotes atherogenic monocyte recruitment through non-cognate engagement of CXCR2 [113]. As discussed in Section 1, intracellular MIF also directly interacts with CSN5 to control the JNK/AP-1 signaling pathway and p27-dependent cell cycle regulation, and inflammatory MIF secretion in endothelial and tumor cells was found to be controlled by CSN5 [90,114]. We recently studied MIF secretion in BM-derived macrophages (BMDMs) from myeloid-restricted *Csn5*-deficient atherogenic mice (*Csn5^Δmyeloid^/Apoe^−/−^*) and found a significant increase in MIF secretion in the absence of Csn5 [91], which implies that Csn5 attenuates monocyte recruitment partially via MIF.

Expanding on this observation, we explored the impact of myeloid-specific *Csn5*-deficiency in experimental atherosclerosis in vivo, using high fat diet-fed atherogenic *Apoe^−/−^* mice as a preclinical model of atherosclerosis. Myeloid *Csn5* deficiency was found to lead to exacerbated atherosclerotic lesion formation across the entire aortic bed, with markedly increased plaque sizes seen in aortic root and arch, as well as thoracic and abdominal aorta, which suggests that CSN5 has a potent athero-protective capacity [91]. Along the same line, BMDMs from *Csn5^Δmyeloid^/Apoe^−/−^* mice produce increased levels of inflammatory cytokines when compared to *Apoe^−/−^* mice expressing normal myeloid CSN5 levels. Conversely, the pharmacologic agent MLN4924, mimicking CSN5 hyperactivity, abrogates inflammatory cytokine and chemokine expression in BMDMs. This effect mirrors the abrogation by MLN4924 of chemokine (such as CCL2) and adhesion molecule (i.e., ICAM, VCAM) expression seen in atherogenic stimulated endothelial cells (see Section 2.1), which, together, suggests that CSN5, and possibly the CSN holo-complex, potently attenuate atherogenic inflammatory responses in two pivotal atherogenic cell types, which are endothelial cells and monocytes/macrophages.

The expression of atherogenic cytokines, chemokines, and adhesion molecules is chiefly regulated by the NF-κB pathway [87]. We, therefore, asked whether the observed anti-atherogenic capacity of CSN5 in myeloid (and endothelial) cells is mediated through an inhibitory effect on NF-κB signaling. Compared to BMDMs from *Csn5*-proficient *Apoe^−/−^* mice, BMDMs from *Csn5^Δmyeloid^/Apoe^−/−^* mice exhibited significantly reduced levels of the inhibitory protein IκB-α under resting conditions and following BMDM stimulation by LPS or TNF-α. Conversely, *Csn5* depletion led to elevated p65 transcriptional activity, whereas MLN4924 blunted p65 activation in inflammatory stimulated BMDMs [91]. Furthermore, the NF-κB pathway-blocking micro-RNAs (miRs) were down-regulated in BMDMs from *Csn5^Δmyeloid^/Apoe^−/−^* mice [91]. Control of inflammatory NF-κB signaling by the CSN as observed in atherogenic ECs and myeloid cells is consistent with observations in cell lines and lymphocytes. As mentioned above, disruption of the CSN by a siRNA-mediated knockdown of individual CSN subunits resulted in a reduced re-accumulation of IκB-α and prolonged nuclear translocation of NF-κB in TNF-α-stimulated cells [47,115]. Additionally, inhibition of NEDDylation by MLN4924 resulted in reduced NF-κB activation in B-cells, myeloid leukemia cells, cervical cancer cells, and macrophages. The stabilizing effect of CSN5 toward IκB-α in stimulated cells was explained both by CSN5-mediated deNEDDylation of cullins, controlling CRL activity, and the association of the CSN with the deubiquitinase USP15 [47,116,117,118].

The NF-κB pathway does not solely control the anti-inflammatory effects of the CSN in monocytes/macrophages. The hypoxia-inducible factor (HIF)-1α is expressed in various cell types present in atherosclerotic lesions, and is associated with lesional inflammation and intra-plaque angiogenesis. It has been reported to either promote or attenuate disease progression [119,120,121,122,123]. CSN5 plays a role in HIF-1α stabilization [124,125,126]. We found that BMDMs from *Csn5^Δmyeloid^/Apoe^−/−^* mice exhibited reduced HIF-1α transcriptional activity [91] and a significant reduction was observed for the HIF-1α target genes Edn1 and Opn1 upon *Csn5*-deletion in LPS-stimulated macrophages.

The potential translational relevance of these findings became clear, when atherogenic *Apoe^−/−^* mice were therapeutically treated with MLN4924, which had previously shown promising results in animal tumor models and in clinical trials for cancer [43,56,118]. In the experimental atherosclerosis model, MLN4924 simulated conditions of CSN5 overexpression in vivo. Of note, it inhibited early atherosclerotic lesion formation in aorta and aortic root as well as acute atherogenic inflammation [91], which confirms that the COP9 signalosome plays a significant role in atherosclerosis progression in vivo and highlights its potential pharmacological capacity in cardiovascular diseases.

### 2.3. The COP9 Signalosome Regulates the Cholesterol Efflux Pathway in Foam Cells

Atherosclerotic lesion development, among other hallmarks, is characterized by intimal macrophage transformation into foam cells and dysregulated lipid turnover of these cells. Foam cells form upon massive uptake of modified lipoproteins (e.g., oxLDL, AcLDL) by plaque macrophages [104,127,128,129]. Homeostasis between the influx and efflux of cholesterol is crucial for foam cell formation. Key mediators for macrophage cholesterol homeostasis in this respect are scavenger receptors such as CD36 or scavenger receptor class A (SRA) and ATP-binding cassette (ABC) transporters [128,130]. 

ABC transporters such as the ATP-binding cassette transporter A1 (ABCA1) are key components of cholesterol efflux pathways [130]. It promotes cholesterol efflux to lipid-free apoA-1 to form high-density lipoproteins (HDL) [131]. Therefore, ABCA1 can be considered an effective tool in preventing foam cell formation from macrophages and to act as an athero-protective mediator. Proteasomal degradation regulated macrophage ABCA1 levels [132] and inhibition of ABCA1 protein degradation increases the cholesterol efflux capacity of an intimal macrophage, which potentially slows down atherosclerosis progression [132]. ABCA1 was found to be more susceptible to ubiquitination-dependent degradation processes under hypercholesterolemic conditions [133,134], which is a notion that may connect ABCA1 turnover to the activity of the COP9 signalosome. In fact, Azuma et al. reported that the CSN is involved in the ubiquitylation and stability of ABCA1 [135]. Subunits CSN2 and CSN5 were found to co-precipitate with ABCA1 when co-expressed in HEK293 cells and overexpression of CSN2 not only led to increased endogenous CSN7 and CSN8 levels, likely by CSN holo-complex stabilization, but also decreased ubiquitinylated forms of ABCA1, which suggests that the CSN promotes ABCA1 stabilization and cholesterol efflux functionality. The accumulation of excess intracellular-free cholesterol causes late lesion-based inflammation mediated by macrophages and the apoptosis of macrophages [136,137]. Since ABCA1 stabilization has been linked with an improved cholesterol efflux rate in macrophages, dysfunction of the CSN might facilitate abnormal ABCA1 degradation in advanced atherosclerotic plaque tissue [132,133].

One study insinuates a mechanism as to how a dysfunction of the CSN in atherosclerotic tissue might arise. The pro-apoptotic cytoplasmic pattern recognition receptor Nod1 was found in a yeast two-hybrid screen to interact with CSN subunits through its caspase activation and recruitment domain (CARD) domain and activation of Nod1 and caspase 8, which is a pro-apoptotic caspase that is activated by TNF during apoptosis. These elements were suggested to cleave CSN6, which is one of the MPN domain-containing CSN subunits that co-controls CSN5 deNEDDylase activity [138]. CSN6 degradation may lead to CSN complex disassembly, which, in turn, may give rise to ABCA1 destabilization and an accumulation of excess free cholesterol. However, while work by Hetfeld and colleagues confirms caspase-mediated CSN6 cleavage under apoptotic conditions, it implicates caspase 3 rather than caspase 8and suggests that this activity promotes CSN-mediated deNEDDylation activity [139], which, while not tested in that study, may potentially counteract ABCA1 stability. The relationship between apoptosis-triggered CSN6 cleavage and ABCA1 stability, thus, needs to be studied further in atherosclerosis-relevant models.

Furthermore, a recent study by Schwarz et al. showed that oxLDL promotes an increase in CSN5 protein expression in differentiated human macrophages. However, CSN5 did not directly regulate macrophage cholesterol content, nor did it influence the NF-κB signaling upon oxLDL stimulation, whereas changes in p38 MAPK were observed in differentiated human macrophages following oxLDL stimulation [140]. 

Together, only a few studies have addressed the role of the CSN in foam cell formation and plaque macrophage homeostasis and it remains unclear how CSN5 and/or the CSN holo-complex affect foam cell-related pathologies.

### 2.4. The COP9 Signalosome May Play a Role in the Proliferation of Vascular Smooth Muscle Cells 

Vascular smooth muscle cells (VSMCs) are the major cell type of the *Tunica media* and regulate vascular tone and blood flow through dynamic cycles in cell contraction and relaxation [141]. In healthy blood vessels, they are quiescent and exhibit low rates of proliferation and are referred as a contractile phenotype VSMCs. However, under pathological conditions, VSMCs undergo a de-differentiation process and phenotypic changes, characterized by decreased contractile marker expression, an increased proliferation rate, enhanced migration, and boosted extracellular matrix synthesis. The proliferative/secretory phenotype of VSMCs has been implicated in the pathophysiology of atherosclerosis, especially accelerated atherosclerosis and neointima formation in stent restenosis, pulmonary hypertension, systemic hypertension, and cancer [142,143,144].

Studies on a role of the COP9 signalosome in VSMC phenotype changes are limited. However, emerging indirect evidence may imply that the CSN modulates VSMC biology and pathobiology. Krüppel-like factor 4 (KLF4), which is a pluripotency transcription factor absent in contractile VSMCs, promotes VSMC dedifferentiation [144]. Krüppel-like factor 4 is up-regulated in vitro in response to platelet-derived growth factor (PDGF)-mediated cholesterol loading and oxidized phospholipid accumulation [145]. Using pulmonary arterial smooth muscle cells (PASMCs), Zhu et al. showed that CSN6 levels are up-regulated by activation of the PDGFR/PI3K/Akt signaling pathway [146]. This was accompanied by a reduction in β-TrCP CRL substrate adapter levels and an increase in Cdc25A, which is a known substrate of SCF^β-TrCP^ CRLs. These changes were reversed by inhibition of the PDGFR/PI3K/Akt signaling pathway with Imatinib or CSN6 downregulation by siRNA approach [146]. This indicated that up-regulation of CSN6 by PDGFR/PI3K/Akt signaling promotes β-TrCP degradation, which, in turn, stabilizes the cell cycle protein Cdc25A and results in PASMC proliferation.

Phenotypic switching of VSMCs requires that they re-enter the cell cycle. Important regulators of this process are cyclin-dependent kinase inhibitors (CKDIs) such as the KIP/CIP family proteins p21, p27, and p57 [147]. As detailed in Section 1, p27 levels are regulated by SCF^Skp2^ CRLs, which, in turn, are controlled by the CSN [43]. Hence, the CSN might control VSMC proliferation through this pathway [148]. However, direct evidence has been elusive. The ET/CSN link might constitute another indirect connection between the COP9 signalosome and pathologic VSMC proliferation, since ET-1 signaling leads to a decrease in p27 expression [149,150,151]. ET-1 also induces long-lasting vasoconstriction, stimulates VSMC proliferation (VSMCs) [152], and has been suggested to play an important role in hypertension, vascular remodeling, cardiac hypertrophy, and coronary artery disease [153]. As discussed above, CSN5 regulates the expression levels of components of the ET receptor/ligand system.

Lastly, the fibroproliferative phenotype of VSMCs might be connected to the activation of the AP-1 pathway by pro-atherogenic oxidized LDL stimulation. Mazière et al. found that oxidative stress induced by oxidized LDL activated AP-1 in SMCs. While they have not directly studied a role of the CSN holo-complex or complex-independent CSN5 in this setting, it could insinuate a link between the CSN and pro-oxidative atherogenic stress via AP-1 [154].

Together, while direct evidence is still missing, several studies suggest that the CSN, via different pathways, might indirectly suppress VSMC proliferation in vascular pathologies.

### 2.5. The COP9 Signalosome Regulates the Cell Cycle and Proliferation of T Cells

T lymphocytes are key players in adaptive immunity and their role in atherosclerosis progression is well established [155]. This comprises various inflammation-promoting effects along the ‘response to injury’ paradigm, their interplay with pro-atherogenic innate immune cells, as well as responses to intra-plaque self-antigens and non-self-antigens that can influence the initiation, progression, and stability of plaques. Pro-atherogenic activity is not only based on Th1 cell activity, but encompasses Th17 effects, while Tregs have been recognized as potent anti-atherosclerotic cells. T cells also shape atherogenic B-cell responses, which, more recently, have been recognized to play an important role in atherogenesis [68,69,156]. 

Conditional deletion of *Csn8* was shown to be essential for peripheral T-cell homeostasis and T-cell receptor-induced entry into the cell cycle from quiescence [157]. Conditional deletion of *Csn8* using CD4-Cre driver mice in peripheral T cells reduced T-cell survival and proliferation in vivo and attenuated antigen-dependent IL-2 production. *Csn8*-deficient T cells were unable to re-enter the cell cycle from the G0 quiescent state and exhibited reduced levels of cell cycle-related genes, but caused an upregulation of the cell cycle inhibitor p21^Cip1^. Since the deletion of *Csn8* interfered with the formation of the CSN holo-complex, it is likely that the observed impaired T-cell activation and defective cell cycle phenotype is a consequence of the impaired CSN holo-complex formation [157], which suggests that the CSN controls transcription in antigen-induced T-cell proliferation. A role of CSN8 in cell cycle control was confirmed in mouse embryonic fibroblasts and HeLa cells. That study suggested that Csn8 is important in maintaining the duration of the G1 phase [158]. 

T-cell progenitors, the thymocytes, go through various maturation steps that differ based on cell surface markers [159]. Early thymocytes lack the expression of the co-receptors CD4 and CD8 and are termed double-negative (DN). Development and differentiation are accompanied by an up-regulation of CD4 and CD8 to eventually give rise to double-positive (DP) thymocytes [160]. *Csn5*-depleted thymocytes show impaired cell cycle progression and massive apoptosis at the DN to 4-DP stage. Moreover, it was shown that genetic deletion of *Csn5* in thymocytes causes a marked reduction in nuclear NF-κB and a decreased expression of anti-apoptotic NF-κB target genes including p53, IκB-α, and β-catenin [115]. 

Therefore, the CSN controls T-cell development and activation in multiple ways, but the significance of these functions in atherosclerosis has not yet been explored directly.

### 2.6. Role of the COP9 Signalosome in Adipocytes

Increasing evidence has revealed that adipose tissue functions as a source of many types of cytokines, called adipocytokines, which modulate glucose metabolism, lipid profiles, and inflammation. Of note, most arteries are surrounded by perivascular adipose tissue (PVAT) [161,162,163,164]. PVAT is not just a structurally supportive tissue component for the vasculature, but recent studies showed that it influences vasodilation and vasocontraction and plays a role in atherogenesis [165]. Moreover, adipocytes control cholesterol homeostasis. Adipose tissue contains the largest pool of free cholesterol in the body and can uptake and degrade oxLDL through e.g., CD36 [166], which is a process that is impaired under hypercholesterolemic conditions [166,167]. After all, there are numerous causal links between obesity, inflammation, and atherosclerotic pathologies [168]. Adipocyte differentiation involves a temporally regulated set of gene-expression events and several transcription factors of the C/EBP family have been found to participate in adipogenesis, including C/EBPα, C/EBPβ, C/EBPγ, C/EBPδ, and CHOP [169]. Both, CSN7A and CSN7B possess 59% protein sequence identity [170,171]. A recent study demonstrated that the CSN plays a role in adipocyte differentiation, with endogenous CSN7B increased and CSN7A and CSN7B down-regulation slowed down in adipogenesis [171]. In fact, most of the key regulators of adipogenesis are targeted by the UPS [172,173] and CHOP is ubiquitinated by the CRL3^Keap1^ E3 ligase complex [174]. Moreover, permanent down-regulation of CSN1 led to a complete block of adipogenesis, which confirms a role for the CSN in adipocyte differentiation [172]. Thus, the CSN is necessary for adipogenesis, but how precisely this may link to atherogenesis has not yet been addressed in in vivo models. 

## 3. The Role of the COP9 Signalosome in Cardiovascular Ischemia 

Cardiovascular diseases including stroke remain the leading cause of death and morbidity worldwide despite advances in interventional and medical treatment [175]. Myocardial infarction (MI)/ischemic heart disease (IHD) is an acute condition leading to ischemic damage of heart tissue and an inflammatory infiltrate into the heart. Additionally, MI triggers long term adverse changes in the heart that have collectively been termed heart failure (HF). The links between the CSN and these long-term adaptation processes after cardiac ischemia such as in heart failure are discussed in Section 4. The present chapter will focus on ischemic stress as it occurs in ischemic stroke. Ischemic stroke is caused by cerebral blood vessel obstruction and is the most common stroke subtype. It makes up for 70% to 80% of recorded stroke cases. Large artery atherosclerotic stroke (LAS) causes almost half of ischemic stroke cases. As discussed in detail above, atherosclerosis is a lipid-triggered chronic inflammatory disease of the arterial vessel wall and is the main underlying cause of not only coronary artery disease (CAD) but also LAS [176]. While several studies in the last five to 10 years have addressed the role of the CSN in atherosclerosis and neointimal hyperplasia (see Section 2), to date, very few studies have directly examined links between the CSN and ischemic stroke. This holds true for studies both on the expression of CSN subunit mRNA or protein in stroke tissue and on a potential causal role that the CSN or one of its subunits may have in stroke. We briefly discuss these studies and their implications.

One report identified subunit CSN5 in the context of a genetic knockout of the inflammatory cytokine MIF that was studied in a mouse model of transient middle cerebral artery occlusion (tMCAO) [176]. In this study, CSN5 was shown to be increased in the mitochondria of females but not male *Mif* knockout mice in the early hours post-stroke. CSN5 was suggested to mediate the neuroprotective activity of intracellular MIF in female stroked mice [176]. MIF is a secreted chemokine-like inflammatory cytokine that generally promotes atherosclerosis and ischemic stroke [177,178,179], but, as discussed in Section 1, due to its atypical expression characteristics, has intracellular roles and has been identified as an intracellular interactor of CSN5 [49]. The MIF/CSN5 interaction was initially thought to occur in the cytosol, but the study by Turtzo et al. suggests an additional link to a mitochondrial location. In addition, it is known that immune response pathways after ischemic stroke vary by sex [180]. Accordingly, Turtzo et al. suggested a sex-specific mitochondrial translocation mechanism for CSN5 in *Mif*-deficient mouse brains after stroke. Specifically, their data may be in line with the following mechanism: (i) in female stroked mouse brains, MIF protects from early ischemic damage by retaining CSN5 in the cytosol, which prevents it from entering mitochondria and blocking CSN5/steroid receptor coactivator (SRC)-mediated transcriptional activation of apoptotic programs, (ii) in the absence of MIF, CSN5 traffics into mitochondria, where it interacts with pro-apoptotic Bcl-G proteins and impairs mitophagy, (iii) in parallel, nuclear CSN5/SRC complexes promote pro-apoptotic transcriptional programs, and (iv) resulting in CSN5-mediated neuronal death in female *Mif-*deficient stroked brain tissue, while the precise mechanism of sex-specificity was not clarified in that study. One explanation could come from two studies reporting that CSN5 binds to the progesterone receptor in addition to SRC to mediate the transcription and/or have a synergistic effect with the transcription factor E2F1 [181,182,183]. Furthermore, a YTH screen showed that mitochondrial CSN5 interacts with Bcl-G proteins to promote apoptosis [184]. 

Following stroke, ischemia usually comes with poor outcomes, whereas ischemic preconditioning (IPC) is a major hypoxia-adaptive pathway for the brain to protect itself from subsequent ischemia [185]. IPC has been found to convey adaptation/protection in several tissues including heart, kidneys, and lung. Inflammatory responses are essential for micro-environmental metabolic changes, and protein degradation mediated via cell signaling pathways (e.g., NF-κB) is a principal feature of inflammation [185]. To this end, the CSN was found to be connected to antiinflammatory adaptation to hypoxia in the context of IPC. siRNA-mediated silencing of *CSN5* partially reversed IPC-mediated inhibition of NF-κB in HeLa cells and lung tissue following stimulation of the Ado A2B adenosine receptor subtype. The study suggested that IPC induces extracellular accumulation of adenosine and suppresses NF-κB activity through deNEDDylation of Cul-1 [186]. In the human microvascular EC line HMEC-1, mimicking overexpression of CSN5 by MLN4924, downregulated TNF-α/LPS-induced proinflammatory cytokine transcription and protein levels, and rescued the increased endothelial permeability, which is, otherwise, seen upon LPS stimulation. In turn, HIF-1α levels and activity were increased by blocking cullin NEDDylation in these cells. To this end, the benefits of HIF stabilization have been shown in mouse models of a number of diseases [187]. For example, the HIF-1α-induced ectonucleotidase CD73, which converts adenosine-monophosphate (AMP) into adenosine, has been shown to be protective in the setting of IPC in the kidney [188]. Similarly, the NF-κB activity inhibitor MLN519 (previously named PS519) inhibits 26S proteasome activity and was found to be neuroprotective in stroke [189]. Various time point and dose studies of MLN519 in an MCAO injury rat model indicated that targeting the delayed NF-κB-regulated neuro-inflammatory response phase resulted in reduced rat brain injury, as well as improved neuro-functional recovery [190,191,192,193]. 

In conjunction, the above discussed studies provide initial hints that the CSN might have a critical role in microvascular diseases including ischemic stroke and that it will be important to explore the role of the CSN in stroke and the underlying mechanisms further.

## 4. The Role of the COP9 Signalosome in Cardiac Proteotoxicity and Heart Failure 

Cardiac disorders are associated with impaired degradation of misfolded proteins. Conversely, protein quality control is essential for maintaining intracellular protein homeostasis in cardiomyocytes and cardiac fibroblasts, and, thereby, eventually for protecting from heart failure (HF). We will first discuss work relating the CSN to cardiac proteotoxicity and then, more specifically, focus on other dysfunctions related to the clinical setting of HF. 

### 4.1. The Role of the COP9 Signalosome in Cardiac Proteotoxicity

The UPS and autophagy are the two main proteolytic pathways in the cell [194,195] and UPS dysfunction has been observed in various forms of heart diseases [196,197,198]. A role of the COP9 signalosome in controlling the ubiquitination of cardiotoxic proteins and autophagosome function in the heart is emerging. However, as discussed in previous chapters, the global genetic deletion of CSN subunits causes premature death, as noted for different CSN subunits in the respective knockout mice [199,200]. Consequently, subsequent approaches aimed at cell-specific conditional and inducible gene targeting strategies of CSN subunits. For the heart, this has, so far, mostly been applied to CSN8, but also CSN 3 and 5 have been studied in cardiomyocytes.

Wang, Wei, and colleagues generated a perinatal cardiomyocyte-restricted *Csn8* gene knockout in mice and established that CSN8 (and the CSN) regulate UPS function in the heart [201]. *Csn8* deficiency in cardiomyocytes led to impaired CSN holo-complex formation and cullin deNEDDylation, accompanied by a decrease in F-box proteins. Moreover, by cross-breeding with mice expressing a transgene of a surrogate misfolded protein (GFP fused to the degron CL1, which features a signature conformation of misfolded proteins and is readily processed by a functional UPS machinery), they showed that *Csn8* deficiency markedly impairs UPS proteolytic function in the heart. Cardiomyocyte-restricted *Csn8^–/–^* mice developed cardiac hypertrophy, which progressed to HF and premature death. This was found to be due to substantial cardiomyocyte necrosis and inflammatory leukocyte infiltrates, which, together, suggest that CSN8 and the CSN play an essential role in UPS-mediated degradation of misfolded cardiac proteins and cardiomyocyte survival [201]. This notion was confirmed using a *Csn8* hypomorphism mouse model. These mice, which exhibit an 80% reduction of Csn8 levels in the heart, while circumventing the problems of premature cell death, were crossed with mice featuring the cardiomyocyte-restricted transgenic (tg) expression of CryAB^R120G^ [202]. CryAB^R120G^ tg mice feature the pathogenic role of cardiac proteotoxicity in desmin-related cardiomyopathy (DRC, DRM). The cardiomyopathy is caused by a mutation in the desmin-associated protein αβ-crystallin (CryAB) [203,204]. The DRM-linked R120G missense mutation of CryAB is a bona fide misfolded cytosolic protein that leads to intra-sarcoplasmic desmin-positive aberrant protein aggregates and disruption of the cytoskeletal network. Using this model, they showed that CryAB^R120G^ is overexpressed in the heart and that CSN8 hypo-morphism aggravates CryAB^R120G^-induced cardiomyopathy. This was accompanied by an enhanced accumulation of protein aggregates, increased NEDDylated proteins, and a reduced overall ubiquitination rate [202]. Together, the studies provided strong evidence for a role of CSN8 and the CSN in promoting the ubiquitination and degradation of misfolded proteins in the heart and in protecting against cardiac proteotoxicity. In addition, CRLs have a regulatory role in the degradation of misfolded cytosolic proteins.

CSN8 is also linked to autophagosomal activity in the heart. Cardiomyocyte-restricted *Csn8* deletion led to an increase in the autophagosome signature proteins LC3-II and p62 [205]. In fact, autophagic flux assessments revealed that defective autophagosome removal underlies autophagosome accumulation and occurs before functional UPS impairment in *Csn8*-deficient hearts. Autophagosome accumulation coincided with a downregulation of Rab7 and cardiomyocyte necrosis in *Csn8*-deficient hearts. Reduced Rab7 levels impaired autophagosome maturation and exacerbated cardiomyocyte death [205]. Furthermore, the autophagosomal phenotype of *Csn8*-deficient hearts of adult mice was linked with increased levels of oxidized proteins, necrotic cardiomyocytes, and morphological and functional changes characteristic of dilated cardiomyopathy [206]. To further understand how *Csn8*-deficiency leads to cardiomyocyte necrosis, a perinatal myocardial transcriptome analysis was performed in another study with a focus on differentially expressed genes and different pathways including the CRL substrate receptors (SRs), chromatin remodeling genes, autophagy, vesicle trafficking, endocytosis, and cell death, which compare homozygous myocardial-restricted *Csn8*-deficient mice with corresponding heterozygous mice and a control group [207]. That study and another one indicated that myocardial CSN8 not only acts through post-translational pathways but also on a transcriptional level [207,208]. Most strikingly, decreases seen in multiple CRL SR proteins were associated with decreased transcript levels and changes in the chromatin remodeling pathway, which suggests that nuclear CSN8 may play a role in the transcriptional regulation of CRL SRs [208]. The role in altering gene expression patterns is in line with studies in *Drosophila melanogaster*, which demonstrated that the CSN4 can act as a transcriptional repressor in embryonal development and that CSN8-induced transcriptional effects can affect various pathways, both directly and indirectly [209]. A transcriptional function was also suggested for CSN4 in another study, which showed that it interacts with the retinoblastoma protein target gene promoters Rbf1 and Rbf2, and regulates transcription through chromatin remodeling [210].

### 4.2. The Role of the COP9 Signalosome in Heart Failure

Heart failure (HF) frequently develops after MI/IHD, but also in response to hypertension or ischemic hypertrophic cardiomyopathy (ICM), and causes high socio-economic costs in Western and developing countries. Factors such as recurrent MI, infarct size, ventricular remodeling, stunned myocardium, and hibernating myocardium influence the development of left ventricular systolic dysfunction with or without clinical HF after MI. After MI, cardiac remodeling is a necessary step to replace the damaged tissue with scars to preserve cardiac stability. Post-MI cardiac remodeling comprises three phases: an inflammatory, a reparative/proliferative, and a healing phase, characterized by (i) a transient inflammatory response triggered by danger-associated molecular patterns (DAMPs) and cytokines released from injured cardiomyocytes, (ii) cardiac fibroblast activation and their differentiation into collagen-producing myofibroblasts, and (iii) matrix crosslinking and myofibroblast elimination, respectively [211,212,213]. Sustained activation of myofibroblasts, especially in un-infarcted but vulnerable areas, accounts for an expansion of the fibrotic core and adverse ventricular remodeling. Balanced post-infarct healing and scar formation are critical to prevent infarct expansion, while adverse ventricular remodeling can be used as a predictor of HF. It remains a great challenge to limit cardiac damage and optimize infarct healing to prevent the development of adverse left ventricular remodeling and HF [214].

Yeast two-hybrid screening (YTH) and coimmunoprecipitation evidence suggested that CSN5 interacts with the II–III linker of the α1c subunit of the L-type calcium channel in rat hearts. This interaction may be independent of its role as deNEDDylase within the CSN holo-complex. Colocalization experiments located the interaction to sarcolemma membranes and transverse tubules of cardiomyocytes [215]. Functional experiments indicated that CSN5 plays a potential role in the regulation of cardiac L-type Ca^2+^ channels [215]. Since these channels are key regulators of the cytoplasmic calcium concentration in cardiomyocytes, guaranteeing the subsequent increase in calcium efflux through sarcoplasmic reticulum (SR) channels and, therefore, normal heart contraction, their dysregulation may cause HF and sudden cardiac death [216]. Moreover, proper integrin activation and stimulation of integrin-related signaling pathways are necessary to prevent cardiac stress, remodeling, and cardiac hypertrophy and fibrosis [217]. Of note, YTH and coimmunoprecipitation evidence suggested that CSN5 binds to β2 integrin and integrin α-V to control c-Jun transcriptional activity [50,218]. Both studies found that integrin signaling engages CSN5 as a prerequisite for CSN5 nuclear translocation. While one study uncovered a link of this pathway to matrix metalloprotease (MMP1) expression and scarring in fibroblasts [218], it remains open whether this also plays a role in adverse cardiac remodeling. Furthermore, CSN3 binds to β1D integrin, which was found in a yeast two-hybrid screen of a human heart library. Binding of CSN3 to β1D integrin led to the hypothesis that CSN3 or the CSN, in general, may translocate to the nucleus under pathological stress conditions to promote the communication between the extracellular matrix (and, thus, cardiac remodeling responses) and transcriptional programming in the heart [219]. 

In conclusion, the CSN guarantees normal heart physiology. This mainly occurs through transcriptional regulation by the CSN and by controlling the degradation of cardiotoxic proteins via the CRL/UPS route and/or autophagy, but protein-protein interactions of some of its individual subunits with key matrix and myocyte signaling pathways may contribute to its important role in the heart. Modulating CSN activity may have a therapeutic benefit in cardiac remodeling and HF.

## 5. Conclusions and Future Perspectives

**Figure 2:**
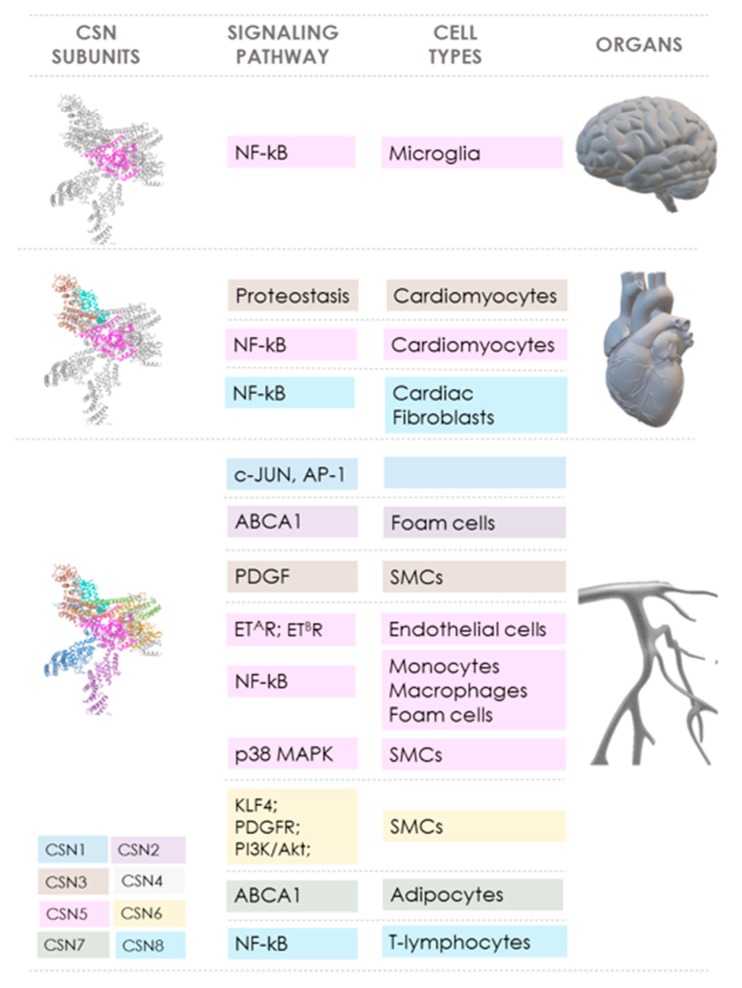
Scheme summarizing the links between COP9 signalosome subunits, signaling pathways, cell types, and organs/tissues in cardiovascular diseases. The cartoon illustrates how CSN (PDB ID: 4D10) subunits CSN1 (dark blue), CSN2 (purple), CSN3 (brown), CSN4 (light gray), CSN5 (pink), CSN6 (yellow), CSN7 (green), and CSN8 (light blue) play a role in cardiovascular diseases. The involved signaling pathways and key regulated proteins as well as the cell types are indicated. The color code of the CSN subunits corresponds to that of the respective signaling pathway and cell type, and is sorted by affected organ (brain (top), heart (middle), and vasculature (bottom)). Details are described in the main manuscript text. Abbreviations: ABCA1, ATP-binding cassette transporter (member 1 of human transporter sub-family ABCA). Akt, protein kinase B. AP-1, activator protein-1. CSN, constitutive photomorphogenesis (COP) 9 signalosome. ET^A^R, endothelin receptor type A. ET^B^R, endothelin receptor type B. MAPK, mitogen-activated protein kinase. NF-κB, nuclear factor kappa-light-chain-enhancer of activated B cells. PDGF, platelet-derived growth factor. KLF4, Krüppel-like factor 4. PDGFR, platelet-derived growth factor receptor. PI3K, phosphatidylinositol 3-kinase. SMCs, smooth muscle cells. Organ images were produced with Microsoft Power Point 2019.

The COP9 signalosome (CSN) controls pivotal cellular pathways both via its role as a key regulator of CRL-dependent protein ubiquitination and degradation processes and through direct activities of some of its subunits. This is the basis for the crucial role of the CSN in cell physiology and homeostasis. It also links the CSN not only to cancer, for which abundant evidence has been gathered over the past 20 years, but, more recently, in vitro and preclinical in vivo data also suggest an emerging role of the CSN in cardiovascular diseases (CVD). Table 1 summarizes the various reported effects of the CSN subunits in CVD, while Figure 2 highlights the signaling pathways and mediators that are controlled by the CSN and the inter-connections between individual CSN subunits, pathways, and organs/CVD disease entity.

Overall, these studies indicate that the CSN protects from CVD and that cardiovascular protection by the CSN is prominently dependent on controlling CRL/UPS pathways in relevant cell types such as cardiomyocytes, endothelial cells, or monocytes/macrophages. Mechanistically, this involves an attenuation of proteotoxicity (in cardiac disease) and the suppression of the inflammatory NF-κB pathway (in atherosclerosis), with most evidence derived from experimental models capitalizing on conditional *Csn8* and *Csn5* gene deficiency, respectively. Various other disease-relevant pathways (i.e., AP-1, HIF-1α) are co-controlled by the CSN and/or its subunits, and these processes are likely to contribute to CSN-mediated cardiac and/or atheroprotection. Evidence for ameliorating activities of upregulated CSN activity in ischemic stroke is still very preliminary, but may involve protection of microvascular barrier integrity and HIF-1α stabilization. Other CVDs such as neointimal hyperplasia and restenosis that are dependent on VSMC dysregulation may be future targets to be addressed.

While CSN-targeting strategies have not yet been tested in clinical trials of CVD, several promising compounds are emerging. These range from compounds such as MLN4924, which mimics CSN activity by preventing cullin NEDDylation and has been successfully tested in phase 1/2 clinical trials for cancer, to subunit-specific inhibitors such as Zn^2+^-chelating JAMM metalloprotease inhibitors or CSN5-specific blockers. 

The various promising studies on the role of the CSN in CVD as summarized in this review article, have established a causal role for the CSN and/or its subunits in CVD and have begun to elucidate the underlying mechanisms. This should encourage future studies to decipher the mechanisms controlled by the CSN in detail, accounting for cell-specific effects and disease phases, and to further explore translational strategies.

## Figures and Tables

**Table 1:** Summary of the role of the COP9 signalosome in cardiovascular diseases.

CSN Subunit	Reported Effects/Activities in Cardiovascular Diseases	References
**CSN1**	CSN1 expression is enhanced during the progression of human atherosclerosis in carotid endarterectomies.	[90]
	CSN1 modulates the JNK/AP-1 pathway	[220,221]
	CSN1 regulates adipogenesis.	[174]
**CSN2**	CSN2 stabilizes the CSN complex and ABCA1 and, therefore, promotes cholesterol efflux functionality.	[136]
	CSN2 promotes JNK/AP-1 signaling.	[221]
**CSN3**	CSN3 and the CSN complex promote cardiac remodeling responses and transcriptional programming in the heart under pathological stress conditions.	[219]
**CSN5**	CSN5 expression correlates with the progression of atherosclerosis in the endothelial layer of human atherosclerotic plaques. It inhibits atherogenic signaling in human and mouse endothelial cells (HUVEC and aortic ECs) by inhibiting NF-κB in primary human and mouse ECs; ‘CSN5 hyperactivity’ (mirrored by MLN4924) abolishes pronounced NF-κB signaling in mouse primary aortic ECs.	[90,91,92]
	CSN5 regulates the expression of components of the endothelin receptor/ligand system by promoting its ubiquitination and degradation associated with increased ERK1/2 phosphorylation.	[103,154]
	CSN5 blocks inflammatory signaling in myeloid cells obtained from atherogenic mice. Csn5 depletion reduces levels of IκB-α and elevates p65 transcriptional activity, and reduces HIF-1α transcriptional activity in myeloid cells from atherogenic mice. ‘CSN5 hyperactivity’ (mirrored by MLN4924) abrogates inflammatory cytokine and chemokine expression in atherogenic myeloid cells.	[91]
	CSN5 impairs the secretion of the inflammatory cytokine/chemokine MIF.	[91]
	OxLDL elevates CSN5 protein levels in differentiated human macrophages possibly via the p38 MAPK pathway.	[140]
	Csn5 regulates cell cycle progression in thymocytes and inhibits apoptosis via the NF-κB pathway in thymocytes.	[48,115]
	CSN5 plays a role in the regulation of normal heart contraction by interacting with cardiac L-type Ca2+ channels. It prevents cardiac stress, remodeling, and cardiac hypertrophy and fibrosis via its interaction with β2 integrin and integrin α-V.	[50,215,218,219]
	CSN5 activates the JNK/AP-1 pathway.	[11]
	CSN5 mediates neuroprotective activity in female *Mif*-deficient stroked brain. ‘CSN5 hyperactivity’ (mirrored by MLN4924) rescues endothelial permeability, down-regulates TNF-α-induced inflammatory cytokine expression, and enhances HIF-α levels in human microvascular ECs.	[176,181,222]
**CSN6**	CSN6 promotes PASMC proliferation via β-TrCP degradation and the cell cycle regulator Cdc25A.	[146]
	CSN6 promotes CSN complex assembly, which enables ABCA1 stabilization and improves cholesterol efflux.	[135,136]
**CSN7**	CSN7A and CSN7B promote adipogenic differentiation.	[171]
**CSN8**	CSN8 expression is elevated during the progression of atherosclerosis in human carotid endarterectomies.	[90,91]
	CSN8 promotes ABCA1 stabilization and cholesterol efflux functionality via CSN holo-complex stabilization.	[135,136]
	CSN8 is essential for the peripheral T-cell homeostasis and T-cell receptor-induced entry into the cell cycle from quiescence.	[157]
	CSN8 and the CSN play an essential role in UPS-mediated degradation of misfolded cardiac proteins and cardiomyocyte survival. CSN8/CSN promotes ubiquitination and degradation of misfolded proteins and protects against cardiac proteotoxicity. *Csn8*-deficient hearts of adult mice are linked with an auto-phagosomal phenotype, increased levels of oxidized proteins, necrotic cardiomyocytes, and dilated cardiomyopathy. CSN8 plays a role in transcription and chromatin remodeling.	[201,202,205,206]

ABCA1, ATP-binding cassette transporter-1. COP9 signalosome, constitutive photomorphogenesis 9 signalosome. EC, endothelial cell. ERK1/2, extracellular signal-regulated kinases-1/2. HIF-1α, hypoxia-inducible factor 1α. HUVEC, human umbilical vein endothelial cell. IκB-α, inhibitor nuclear factor of kappa light polypeptide gene enhancer in B-cells inhibitor-α. MIF, macrophage migration-inhibitory factor. MLN4924, Pevonedistat, protein neddylation inhibitor. NF-κB, nuclear factor kappa-light-chain-enhancer of activated B cells. OxLDL, oxidized low-density lipoprotein. PASMC, pulmonary arterial smooth muscle cell. TNF-α, tumor necrosis factor-α. β-TrCP1, β-transducin repeat-containing E3 ubiquitin protein ligase-1.
